# Different responses of the rhizosphere microbiome to *Verticillium dahliae* infection in two cotton cultivars

**DOI:** 10.3389/fmicb.2023.1229454

**Published:** 2023-08-11

**Authors:** Zhanjiang Tie, Peng Wang, Weijian Chen, Binghui Tang, Yu Yu, Zheng Liu, Sifeng Zhao, Faisal Hayat Khan, XueKun Zhang, Hui Xi

**Affiliations:** ^1^College of Agriculture, Shihezi University, Shihezi, Xinjiang, China; ^2^Xinjiang Academy of Agricultural Reclamation Sciences, Shihezi, Xinjiang, China; ^3^Cotton Research Institute, Shihezi Academy of Agricultural Sciences, Shihezi, Xinjiang, China

**Keywords:** cotton, *Verticillium dahliae*, rhizosphere microbiome, 16S rRNA, ITS

## Abstract

Verticillium wilt is a disastrous disease caused by *Verticillium dahliae* that severely damages the production of cotton in China. Even under homogeneous conditions, the same cotton cultivar facing *V. dahliae* tends to either stay healthy or become seriously ill and die. This binary outcome may be related to the interactions between microbiome assembly and plant health. Understanding how the rhizosphere microbiome responds to *V. dahliae* infection is vital to controlling Verticillium wilt through the manipulation of the microbiome. In this study, we evaluated the healthy and diseased rhizosphere microbiome of two upland cotton cultivars that are resistant to *V. dahliae*, Zhong 2 (resistant) and Xin 36 (susceptible), using 16S rRNA and ITS high-throughput sequencing. The results showed that the healthy rhizosphere of both resistant cultivar and susceptible cultivar had more unique bacterial ASVs than the diseased rhizosphere, whereas fewer unique fungal ASVs were found in the healthy rhizosphere of resistant cultivar. There were no significant differences in alpha diversity and beta diversity between the resistant cultivar and susceptible cultivar. In both resistant cultivar and susceptible cultivar, bacterial genera such as *Pseudomonas* and *Acidobacteria bacterium LP6*, and fungal genera such as *Cephalotrichum* and *Mortierella* were both highly enriched in the diseased rhizosphere, and *Pseudomonas* abundance in diseased rhizospheres was significantly higher than that in the healthy rhizosphere regardless of the cultivar type. However, cultivar and *V. dahliae* infection can cause composition changes in the rhizosphere bacterial and fungal communities, especially in the relative abundances of core microbiome members, which varied significantly, with different responses in the two cotton cultivars. Analysis of co-occurrence networks showed that resistant cultivar has a more complex network relationship than susceptible cultivar in the bacterial communities, and *V. dahliae* has a significant impact on the bacterial community structure. These findings will further broaden the understanding of plant-rhizosphere microbiome interactions and provide an integrative perspective on the cotton rhizosphere microbiome, which is beneficial to cotton health and production.

## Introduction

Under heterogeneity of local conditions, such as the genetic background and abundance of pathogens (Campbell, 1985; Genin and Denny, 2012), host genotypes (Kwak et al., 2018), soil or plant-related microbiome, and soil physicochemical properties (Wei et al., 2019), plants facing many pathogens either stay healthy or become seriously ill and die. However, even under homogeneous conditions, plants facing soil-borne pathogens often exhibit binary outcomes. This binary outcome may be the result of early differentiation of the rhizosphere microbiome, which may further lead to different disease inhibition abilities (Gu et al., 2022).

The rhizosphere is a hotspot where plants exchange substances and energy with their surrounding environment, which serves as the first line of defense against various biotic and abiotic stresses (Li et al., 2020, 2022). Therefore, the rhizosphere microbiome is critical to plant growth and health and is considered the second genome of plants (Mendes et al., 2011). The structure of the rhizosphere microbial community is the result of a series of complex interactions between the plant and soil microbiome (Zhalnina et al., 2018), and its composition and function change during plant development. In addition, the composition of the rhizosphere microbial community is influenced by host genotypes, plant growth stages, climate, microbial species pool, soil types, and agricultural management strategies (Berendsen et al., 2012; Gao et al., 2021). In natural ecosystems where roots and rhizosphere microbiomes coevolve over a long period of time, host genotypes have a great effect on microbial communities (Philippot et al., 2013). Disease-resistant cultivars of bean can resist the invasion of pathogens by enriching specific groups of bacteria in the rhizosphere (Mendes et al., 2017). *Flavobacterium* is significantly enriched in the rhizosphere of resistant tomato cultivars, which could change the rhizosphere microbial community to improve resistance to *Pseudomonas solanacearum* (Kwak et al., 2018). Apart from these factors, pathogen invasion has a great influence on species composition and community diversity (Carrion et al., 2019; Gao et al., 2021) and often occurs in conjunction with changes in diversity and function in the rhizosphere microbiome (Wei et al., 2018; Yuan et al., 2018; Shi et al., 2019), as the function and assembly of the rhizosphere microbiome are tightly coupled (Xun et al., 2019; Luan et al., 2020).

China is the world’s largest cotton producer, accounting for more than 23% of the world’s total output (Meyer and Dew, 2023). However, Verticillium wilt is a kind of plant pathogen with important economic significance that can severely restrict the yield and quality of cotton in China, which can occur during the growing season of cotton, and severe outbreaks can result in yield losses of more than 50% (Ranga et al., 2020; Zhu et al., 2023). Gaining insight into the rhizosphere microbiome’s response to *V. dahliae* may contribute to developing environmentally friendly *V. dahliae* control strategies. In this study, the rhizosphere microbiome of two cotton cultivars with different levels of resistance to *V. dahliae* was investigated using 16S rRNA and ITS high-throughput sequencing. We aimed to (i) assess the effects of *V. dahliae* on the bacterial and fungal rhizosphere microbiome of two cotton cultivars and (ii) determine the differences between healthy and diseased cotton rhizosphere microbiomes.

## Materials and methods

### Experimental design and sample preparation

In this study, two cultivars of upland cotton with different levels of resistance were selected: Zhongzhimian 2 (Zhong 2, resistant to *V. dahliae*) and Xinluzao 36 (Xin 36, highly susceptible to *V. dahliae*). Two cultivars were cultivated in a random arrangement in the Verticillium wilt disease nursery at the Shihezi Academy of Agricultural Sciences, Xinjiang. The field has a continuous cotton growing history of more than 20 years, with a serious and uniform incidence of Verticillium wilt. In April 2021, the seeds of two cultivars were sown in the field, and 18 plants (9 healthy +9 diseased plants for each cultivar) were randomly uprooted with shovels when plants infected with *V. dahliae* showed obvious disease symptoms in August 2021 (Supplementary Figure 1). The disease index (DI) was used to evaluate the severity of cotton Verticillium wilt (Zhang et al., 2012) using the following formula: DI = [Σ (disease grades × number of infected plants)/(total checked plants×4)] × 100 (Zhang et al., 2017).

To collect the rhizosphere soil (1–2 mm–thick soil layer surrounding the root after shaking vigorously), the roots were transferred into a 50 mL centrifuge tube containing 15 mL of 1× phosphate buffer solution (PBS), rotated for 5 min and then removed. Next, the tubes were centrifuged at 4000 × *g* and 4°C for 10 min, and the supernatant was discarded. Then, the samples were centrifuged at 8000 × *g* for 5 min, the supernatant was discarded again, and the remaining part was regarded as the rhizosphere soil (Edwards et al., 2018).

### Microbiome sample collection, PCR amplification and sequencing

Total DNA of soil was extracted from 36 rhizosphere soil samples according to the instructions using the DNeasy PowerSoil Kit (QIAGEN, Germany). The full-length bacterial 16S rRNA gene and fungal ITS were amplified by PCR using the bacteria-specific primer pair 27F (5′-AGAGTTTGATCMTGGCTCAG-3′)/1492R (5′-ACCTTGTTACGACTT-3′) (Thies, 2007) and the fungi-specific primer pair ITS1F (5′-CTTGGTCATTTAGAGGAAGTAA-3′)/LR3 (5′-CCGTGTTTCAAGACGGG-3′) (Kurtzman and Robnett, 1998), respectively. PCR procedure: 95°C for 2 min; 30 cycles of 95°C for 30 s, 55°C for 30 s, 72°C for 30 s with a final extension of 72°C for 5 min. PCR products were purified by Gel Extraction Kit (OMEGA, USA). Then, the entire 16S rRNA gene and ITS lengths of the community were determined using the PacBio Sequel platform at Personalbio, Inc. (Shanghai, China).

### Statistical methods

The sequence data were verified using Quantitative Insights Into Microbial Ecology 2 (QIIME2) and the R software package (version 3.2.0). The QIIME package^1^ was used to extract the high-quality sequences, which were then clustered into amplicon sequence variants (ASVs). Taxonomic assignment of 16S rRNA gene and ITS fragment representative sequences was performed based on the Greengenes database (McDonald et al., 2012) and the UNITE database (Abarenkov et al., 2010). Alpha-diversity analyses included Shannon, Chao1, Simpson, Pielou_e and Observed_species. Beta diversity was calculated by the weighted UniFrac distance and then analyzed by principal coordinate analyses (PCoA) (Lozupone and Knight, 2005). The Kruskal–Wallis test and permutational multivariate analysis of variance (PERMANOVA) with 999 random permutations were used to analyze significant differences in alpha diversity and beta diversity (Anderson, 2001). Venn diagrams were implemented online to show unique and shared ASVs.^2^ The abundances of healthy and diseased rhizospheres in two cotton cultivars were statistically compared at different taxonomic levels and visualized by histogram and heatmap. Two-sided analysis of variance with a *t* test was used for two-group comparison analyses using STAMP (v.2.0.0) (Parks et al., 2014). Co-occurrence network analysis was conducted at the genus level based on Spearman correlation with a threshold of |r| > 0.6 (*p* < 0.05).

## Results

### Diversity and structure of the rhizosphere microbiome in response to *Verticillium dahliae* infection in two cotton cultivars

A total of 433,283 high-quality bacterial 16S rRNA reads and 461,403 fungal internal transcribed spacer [ITS] reads were obtained via the PacBio Sequel platform from 18 healthy and 18 diseased samples, respectively. These reads were aggregated into 8,034 bacterial ASVs and 1,667 ITS fungal ASVs.

**Figure 1:**
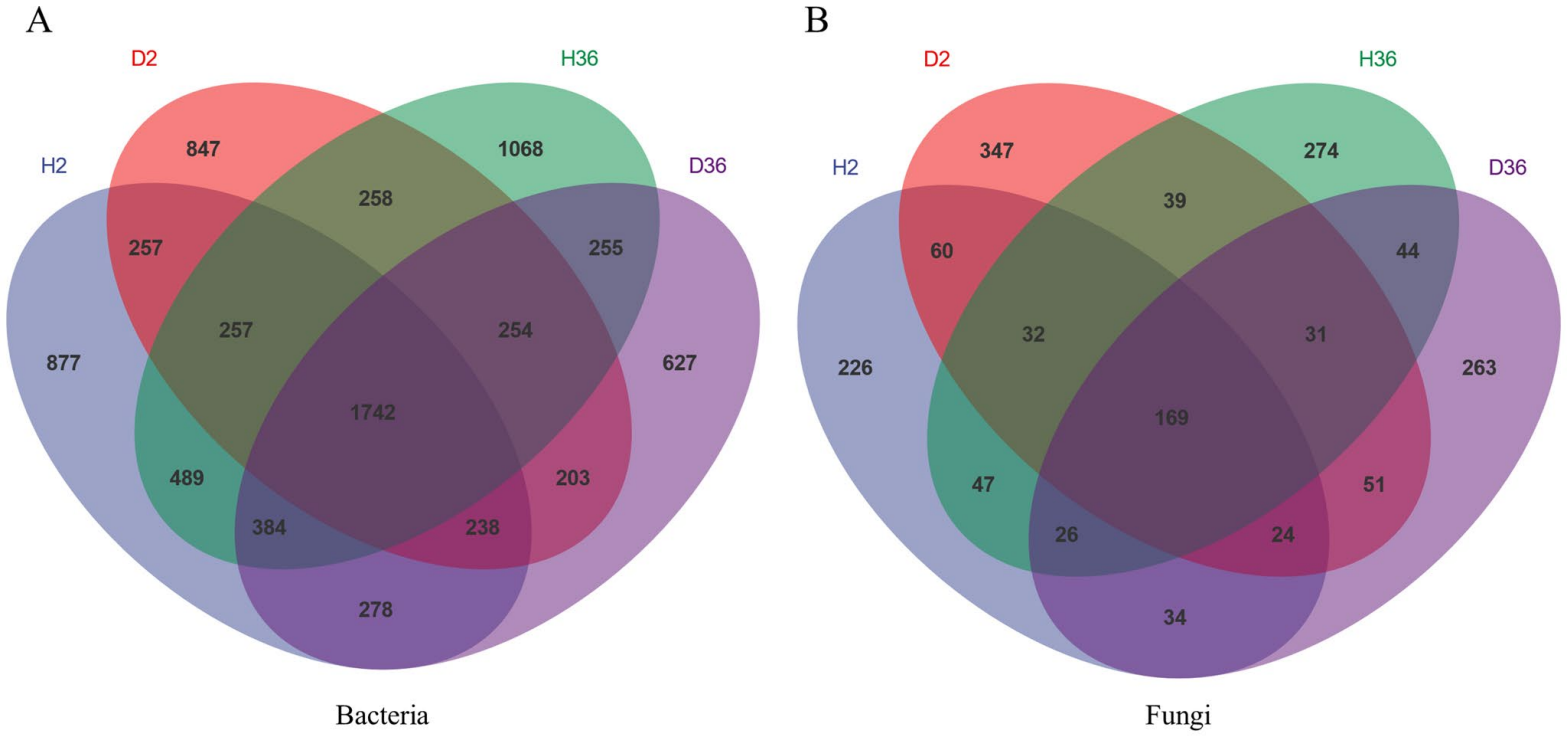
Unique and shared ASVs in healthy and diseased rhizosphere microbiomes of two cotton cultivars.

Venn diagrams show the unique and shared ASVs in the different samples in Figure 1. A total of 1742 bacterial ASVs and 169 fungal ASVs were common to all groups (Figures 1A,B). The healthy rhizosphere of Xin 36 had more unique bacterial (H36, 2072; D36, 1346) and fungal ASVs (H36, 392; D36, 372) than the diseased rhizosphere; the healthy rhizosphere of Zhong 2 had more unique bacterial ASVs than the diseased rhizosphere (H2, 2028; D2, 1562) but fewer unique fungal ASVs than the diseased rhizosphere (H2, 333; D2, 468).

**Figure 2:**
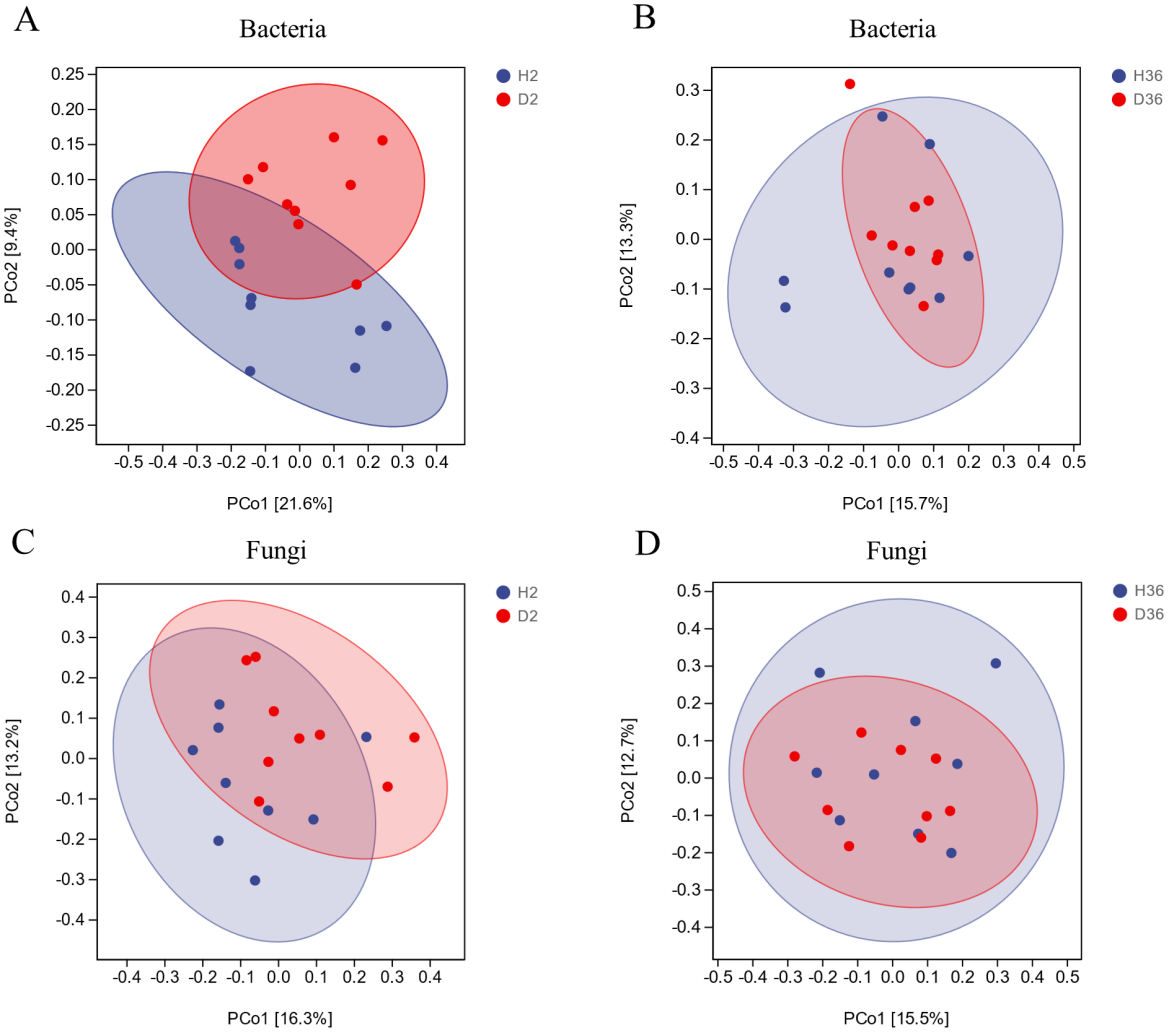
PCoA based on Bray–Curtis distance between healthy and diseased rhizosphere microbiomes of two cotton cultivars (*n* = 36).

Notably, there was no significant difference in alpha diversity between healthy and diseased rhizospheres of both bacteria and fungi in the two cotton cultivars (Supplementary Figure 3). Principal coordinate analysis (PCoA) was performed based on Bray–Curtis dissimilarity and revealed that bacterial and fungal communities showed no significant difference between healthy and diseased rhizospheres, especially for Xin 36 (Figure 2).

### Comparison of rhizosphere community composition between two cotton cultivars

**Figure 3:**
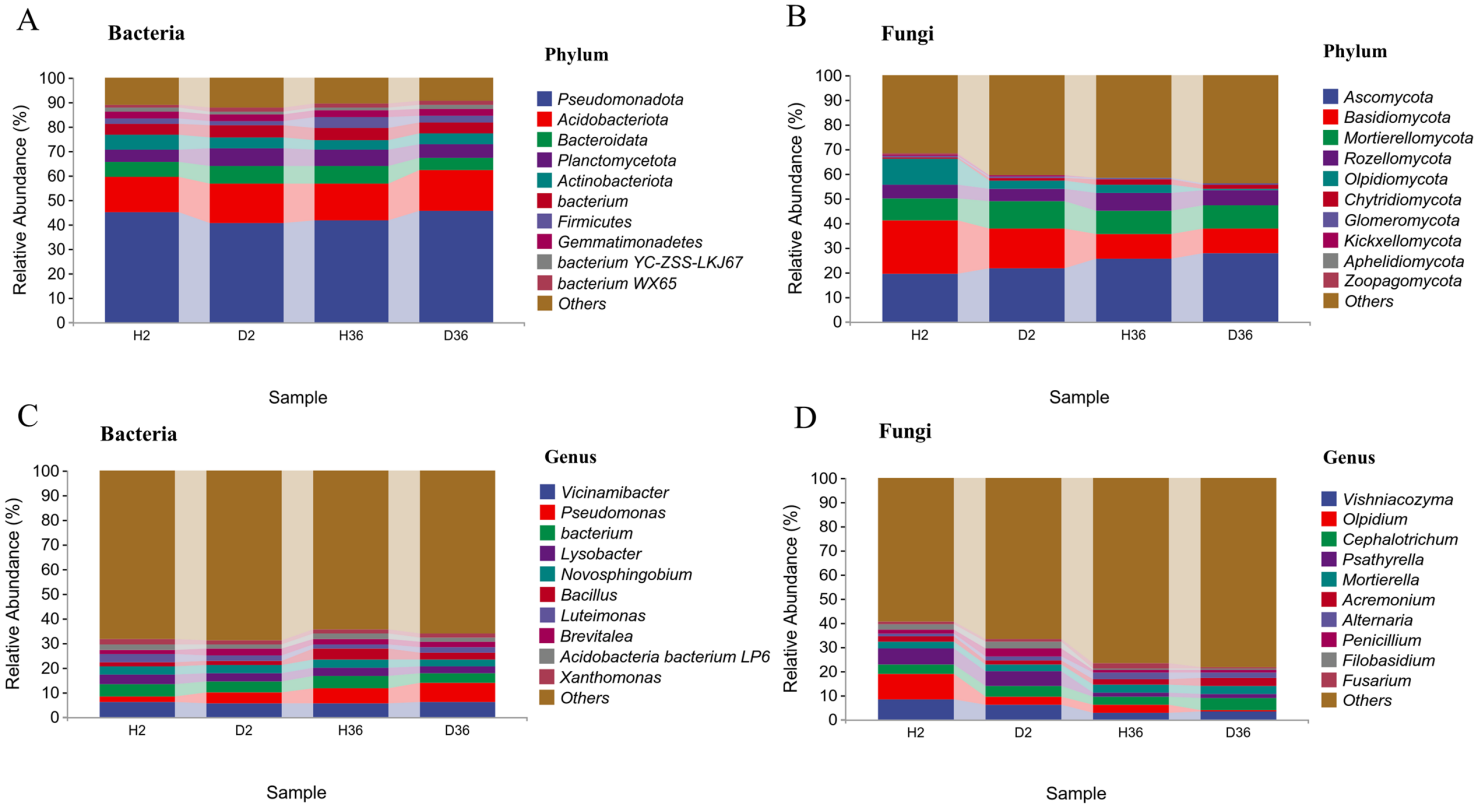
Comparison of the compositions between healthy and diseased rhizosphere microbiomes of two cotton cultivars at the phylum and genus levels.

A total of 28 phyla, 72 classes, 133 orders, 266 families, and 505 genera were identified in the bacterial community. In Zhong 2 and Xin 36, the dominant bacterial phyla were *Pseudomonadota* (42.91%), *Acidobacteriota* (15.78%), *Bacteroidota* (6.34%) and *Planctomycetota* (6.18%) (relative abundance ≥5%), accounting for 71.22% (Figure 3A). At the genus level, most of the bacterial ASVs in Zhong 2 were assigned to *Vicinamibacter* (5.51%), *Bacterium* (4.69%), *Pseudomonas* (3.54%), *Lysobacter* (3.39%) and *Novosphingobium* (3.50%), whereas bacterial ASVs in Xin 36 were mainly classified into *Pseudomonas* (6.84%), *Vicinamibacter* (5.69%), *Bacterium* (4.48%), Bacillus (3.40%) and *Lysobacter* (3.24%) (Figure 3C).

A total of 19 phyla, 52 classes, 94 orders, 160 families, and 219 genera were identified in the fungal community. In Zhong 2 and Xin 36, the dominant fungal phyla were *Ascomycota* (23.67%), *Basidiomycota* (14.28%), *Mortierellomycota* (9.69%), and *Rozellomycota* (6.04%), accounting for 53.68% (Figure 3B). At the genus level, most of the fungal Zhong 2 ASVs were assigned to *Vishniacozyma* (7.10%), *Olpidium* (6.90%), *Psathyrella* (6.38%) and *Cephalotrichum* (4.19%), whereas fungal ASVs in Xin 36 were mainly classified into *Vishniacozyma* (3.06%), *Cephalotrichum* (4.27%), and *Mortierella* (3.42%) (Figure 3D).

**Figure 4:**
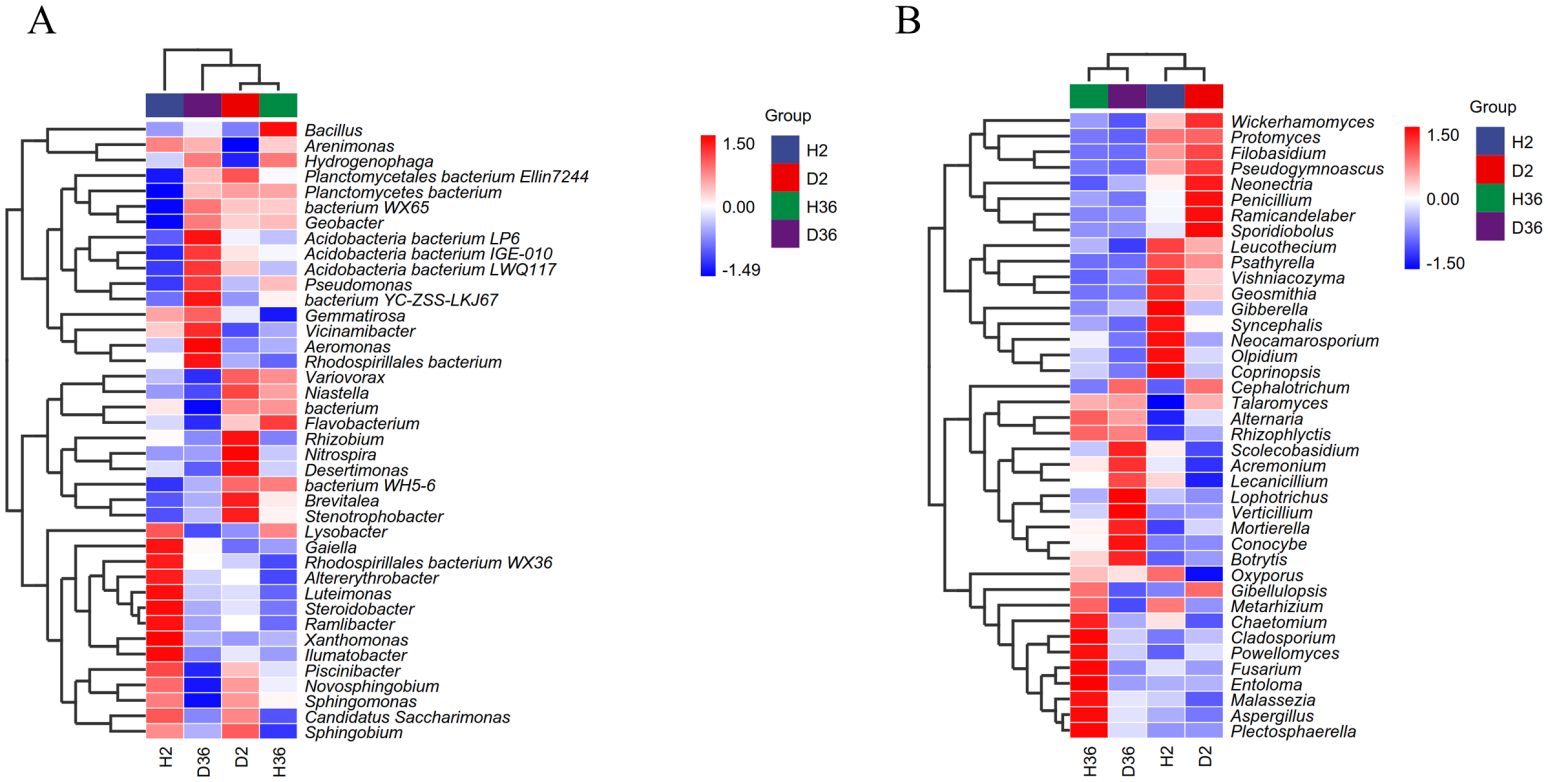
Heatmap analysis of rhizosphere bacterial **(A)** and fungal **(B)** communities at the genus level. The average relative abundance of the genus in the top 40 and *p* < 0.05 were used as the screening criteria, *n* = 36.

Based on the heatmaps of the top 40 genera, the differences between the healthy and diseased rhizosphere microbiomes of the two cotton cultivars were compared (Figures 4A,B). According to the relative abundances of these genera, D2 and H36 were clustered together in the bacterial community, followed by D36 and H2, whereas H2 and D2, and H36 and D36 were clustered together in the fungal community.

### Effects of *Verticillium dahliae* on the composition of rhizosphere microbiomes in two cotton cultivars

**Figure 5:**
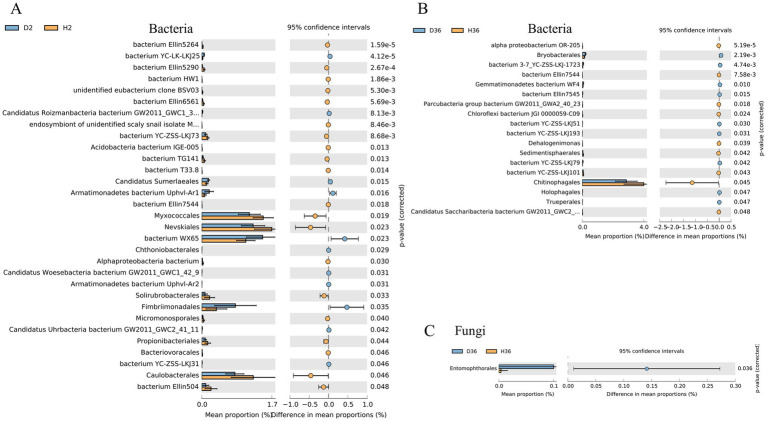
Quantification of the abundance of differential bacterial orders between healthy and diseased rhizospheres of two cotton cultivars using a two-sided *t* test. The corrected *p* values are shown.

We further identified the changes between two cultivars in the taxonomic composition of the rhizosphere microbiomes of the two cultivars. Specifically, the relative abundance of 31 and 18 bacterial orders differed significantly in Zhong 2 (Figure 5A) and Xin 36 (Figure 5B) between healthy and diseased rhizospheres, respectively. In Zhong 2, the relative abundances of bacterium WX65 and Fimbriimonadales significantly increased in D2, while the relative abundances of Caulobacterales, Myxococcales and Nevskiales were significantly reduced. Only Chitinophagales decreased significantly in D36. Furthermore, the relative abundance of Entomophthorales in the fungal order differed significantly in Xin 36 between the healthy and diseased rhizospheres (Figure 5C), and there was no significant change in Zhong 2.

**Figure 6:**
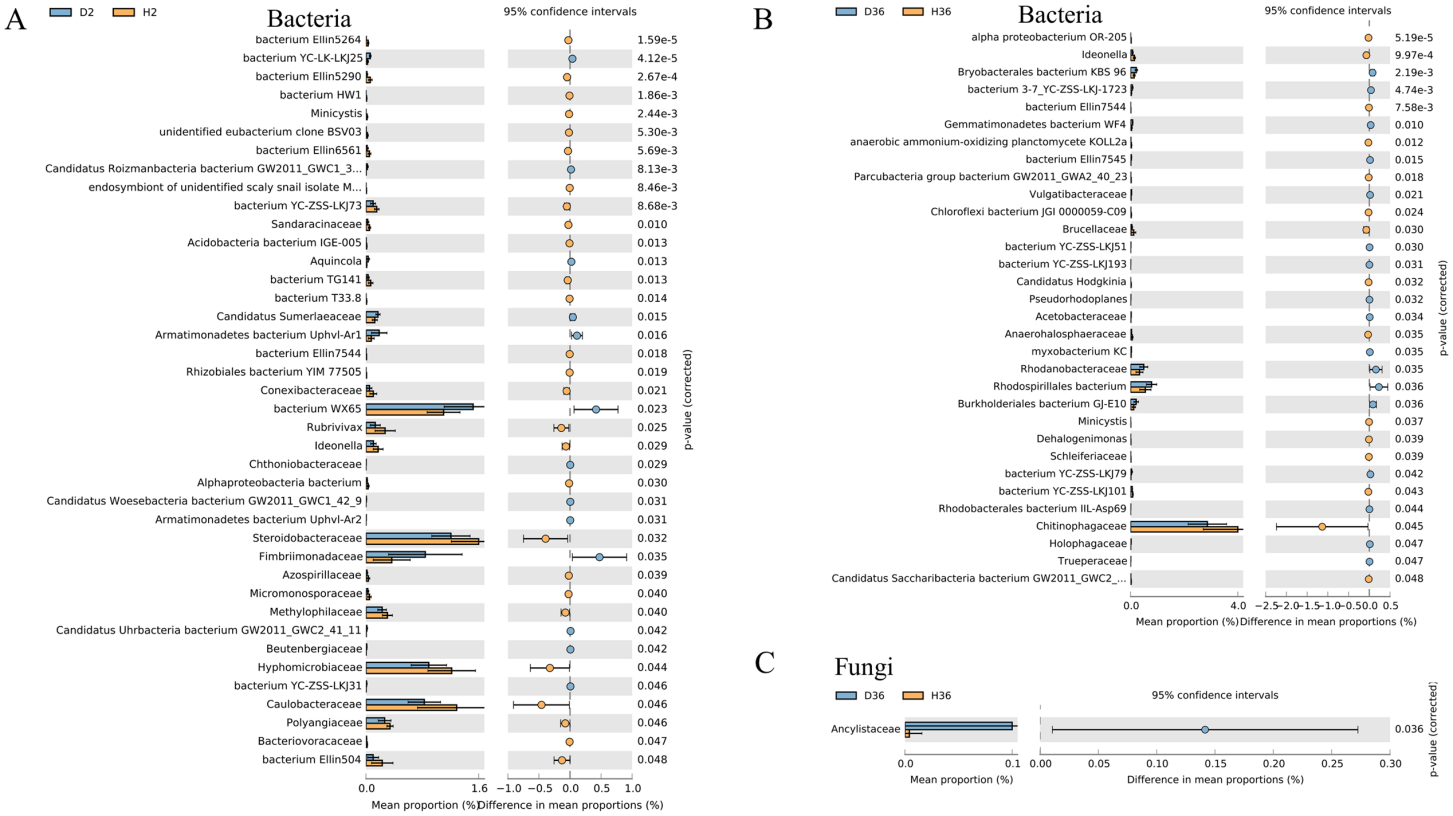
Quantification of the abundance of differential bacterial families between healthy and diseased rhizospheres of two cotton cultivars using a two-sided *t* test. The corrected *p* values are shown.

The relative abundance of 40 and 32 bacterial families differed significantly in Zhong 2 (Figure 6A) and Xin 36 (Figure 6B) between healthy and diseased rhizospheres, respectively. In Zhong 2, the relative abundance of bacterium WX65 significantly increased in D2, whereas the relative abundances of Caulobacteraceae, Hyphomicrobiaceae and Steroidobacteraceae were significantly decreased. Only Chitinophagaceae decreased significantly in D36. Furthermore, the relative abundance of Ancylistaceae in the fungal family differed significantly in Xin 36 between the healthy and diseased rhizospheres (Figure 6C), and there was no significant change in Zhong 2. These results indicated that *V. dahliae* infection influenced the distribution and composition of the rhizosphere bacterial and fungal communities of the two cotton cultivars.

### Different responses of two cotton cultivars to *Verticillium dahliae* infection

The relative abundance of *Verticillium* was significantly different between healthy and diseased rhizospheres of the two cotton cultivars (*p* < 0.05), with high enrichment in diseased rhizospheres compared with healthy rhizospheres. Notably, in both healthy and diseased rhizospheres, the relative abundance of *Verticillium* was higher in Xin 36 than in Zhong 2 (Supplementary Figure 2A). *Verticillium dahliae* and *Verticillium albo-atrum* are potential pathogens causing cotton Verticillium wilt according to previous studies (Qin et al., 2006, 2008). In the genus *Verticillium*, only *Verticillium dahliae* was identified, and the relative abundance was D36 (0.86%) > H36 (0.14%) > D2 (0.03%) > H2 (0%). This was consistent with the phenotypic results of the field Verticillium wilt investigation (Supplementary Figure 2B).

**Figure 7:**
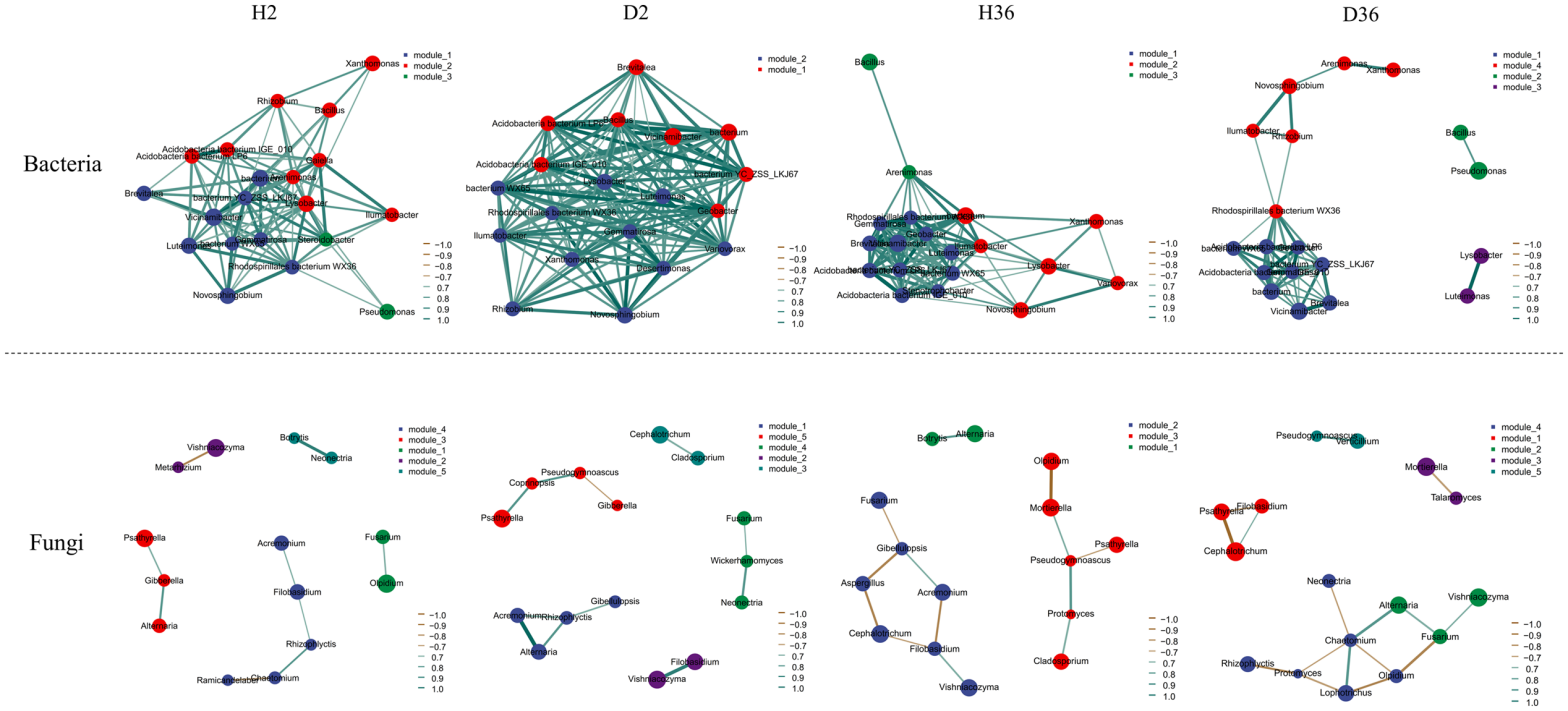
Co-occurrence network analysis of rhizosphere bacterial and fungal communities between healthy and diseased samples in two cotton cultivars. *p* < 0.05, |r| > 0.6.

To determine the effects of *V. dahliae* on rhizosphere microbial co-occurrence patterns between healthy and diseased samples in two cotton cultivars, the genera with the top 20 relative abundances were screened to construct the networks based on correlation relationships (Figure 7). For bacterial communities, the microbial networks in Zhong 2, neither healthy nor diseased rhizospheres, were denser and had a more complex network than those of Xin 36. In contrast, Xin 36 had a more complex network than Zhong 2 in the fungal communities. The results clearly show that the resistance of cotton cultivars has an effect on the complexity of the rhizosphere microbiome. In addition, the influence of *V. dahliae* infection on the complexity of rhizosphere bacterial communities was greater than that of fungal communities.

## Discussion

Rhizosphere microorganisms play an important role in the growth and development of plants, and much research on the plant–microbe and microbe–microbe interactions has been reported in the plant rhizosphere (Lu et al., 2018; Ge et al., 2023). The rhizosphere microbiome is considered to be the first line of defense against soil-borne pathogen infection and abiotic stress, which is vital to the health of plants (Mendes et al., 2013; Ahmed et al., 2022). The balance in the rhizosphere during normal plant growth is disturbed under stress conditions, leading to changes in the composition of the rhizosphere community (Qian et al., 2018). However, there are few studies on how rhizosphere microorganisms of different resistant cultivars of cotton respond to *V. dahliae* infection in the natural field. In this study, we analyzed the different responses of rhizosphere microbial communities in two upland cottons with opposite resistance to Verticillium wilt. The results indicated that *V. dahliae* infection and cultivar alter the composition of the rhizosphere communities, with different responses in two cotton cultivars.

### *Verticillium dahliae* infection influences the structure of the rhizosphere microbial community

After *V. dahliae* infection, the unique bacterial and fungal ASVs in the healthy rhizosphere of Xin 36 were higher than those in the diseased rhizosphere, whereas the healthy rhizosphere of Zhong 2 had more unique bacterial ASVs and fewer unique fungal ASVs than the diseased rhizosphere. The results suggested that *V. dahliae* infection may alter the structure of the rhizosphere microbiome, which is consistent with the result that pathogen infection disrupts host control over the rhizosphere microbiome (Wei et al., 2018; Wen et al., 2020).

The occurrence of diseases is usually accompanied by diversity changes in the rhizosphere microbiome (Wei et al., 2018; Yuan et al., 2018; Shi et al., 2019). However, the analysis results of the alpha and beta diversity showed that there were no significant differences between the resistant cultivar and susceptible cultivar, consistent with a previous study (Fernández-González et al., 2020). Such results may be due to differences in plant host, pathogen, soil, agricultural practices, or environmental conditions (Kwak et al., 2018; Gu et al., 2020; Jiang et al., 2021).

### *Verticillium dahliae* infection shifts the composition of rhizosphere microbial communities

This study demonstrated that the relative abundance of many rhizosphere microorganisms in the healthy rhizosphere differed from that in the diseased rhizosphere of each cultivar. The taxonomic composition of the rhizosphere bacterial communities showed that *Pseudomonadota*, *Acidobacteriota*, *Bacteroidota* and *Planctomycetota* were dominant in two cotton cultivars, with *Pseudomonadota* members accounting for 42.91% of the community composition (Trivedi et al., 2020). Zhong 2 was characterized by a higher relative abundance of *Actinomycetota* than Xin 36, which is known as a biocontrol microorganism (Lee et al., 2021). In addition, the rhizosphere fungal community is mainly composed of *Ascomycota* and *Basidiomycota*, which are the most abundant phyla observed in previous studies (Bálint et al., 2015; Coleman-Derr et al., 2016).

Compared with the rhizosphere microbial communities in the healthy rhizosphere of Zhong 2 and Xin 36, *Pseudomonas* and *Acidobacteria bacterium LP6* in the bacterial communities and *Cephalotrichum* and *Mortierella* in the fungal communities were both highly enriched in the diseased rhizosphere. *Pseudomonas* abundance in diseased rhizospheres was significantly higher than that in the healthy rhizosphere regardless of the cultivar type. Consistently, a previous study found that *Rhizoctonia solani* invasion alters the rhizosphere microbial community and specifically accumulates beneficial *Pseudomonas* (Yin et al., 2021). Therefore, it is reasonable to speculate that the increase in *Pseudomonas* in diseased rhizospheres may contribute to the potential resistance of their host plants to Verticillium wilt. These results suggest that plants may increase the enrichment of specific microbiomes in response to pathogen infection (Busby et al., 2016), which can be used as antagonistic candidates for Verticillium wilt and need to be confirmed by further culture-based experiments.

### Effects of cultivar resistance on the microbial community structure in the cotton rhizosphere

Different microorganisms are recruited by plants to shape their rhizosphere microbiome, and the rhizosphere microflora community structure of the same species changes due to genotype differences (Bressan et al., 2009; Zhang et al., 2021; Yue et al., 2023). The growth and development of different blueberry cultivars were enhanced by recruiting specific rhizosphere microflora based on genotype (Jacoby et al., 2017). Our results showed that the different genotypes of cotton recruited specific rhizosphere microbiomes, suggesting that the rhizosphere microbial community was regulated by host genotypes, consistent with the results in soybean, rice, and barley (Bulgarelli et al., 2015; Singh et al., 2022; Qu et al., 2023). In addition, resistant cultivars may resist pathogen invasion by enriching specific bacterial or fungal groups in the rhizosphere (Mendes et al., 2017; Kwak et al., 2018). The analysis of co-occurrence networks also showed that the resistant cultivar Zhong 2 has a more complex network than the susceptible cultivar Xin 36 in the bacterial communities, and *V. dahliae* has a significant impact on the bacterial community structure compared with fungal communities.

## Conclusion

Analyzing the rhizosphere microbial communities of different resistant cotton is conducive to elucidating the interaction mechanism between cotton and *V. dahliae*, which plays an important role in the green and durable control of cotton Verticillium wilt. In the present study, the healthy and diseased rhizosphere microbiome communities were compared between Zhong 2 and Xin 36, which have significant differences in resistance to *V. dahliae*. The results showed that no significant differences were found in alpha diversity and beta diversity between healthy and diseased rhizospheres in the two cotton cultivars. *V. dahliae* infection and cultivar alter the composition of the rhizosphere communities, with different responses in two cotton cultivars. *V. dahliae* invasion may specifically accumulates beneficial microbiomes, such as *Pseudomonas*, which can be used as antagonistic candidates for Verticillium wilt. Additionally, cultivar and Resistant cultivar has a more complex network relationship than susceptible cultivar in the bacterial communities, and *V. dahliae* has a significant impact on the bacterial community structure. This study analyzed the resistance mechanism of cotton from the perspective of microbiology, and the research results will provide a theoretical basis for the green control strategy of cotton Verticillium wilt.

## Data availability statement

The datasets presented in this study can be found in online repositories. The names of the repository/repositories and accession number(s) can be found below: NCBI database, under accessions PRJNA980128 and PRJNA980194.

## Author contributions

HX and XZ planned and designed the research and experiments. ZT, PW, WC, BT, HX, and XZ performed the experiments. HX analyzed the data. ZT, HX, and XZ wrote the manuscript. HX, ZL, YY, and SZ acquired the funds for the study. All authors have read and approved the final manuscript.

## Funding

This work was supported by the Shihezi University high-level talents research project (project number RCZK202016), National key research and development plan project (project number 2022YFD1400305-02), Basic research project of the Corps (project number 2023CB007-08), and Major science and technology special projects of Autonomous region (project number 2022294083).

## Conflict of interest

The authors declare that the research was conducted in the absence of any commercial or financial relationships that could be construed as a potential conflict of interest.

## Publisher’s note

All claims expressed in this article are solely those of the authors and do not necessarily represent those of their affiliated organizations, or those of the publisher, the editors and the reviewers. Any product that may be evaluated in this article, or claim that may be made by its manufacturer, is not guaranteed or endorsed by the publisher.

## Footnotes

^1^http://qiime.org/acripts/pick_oyus.html

^2^http://bioinformatics.psb.ugent.be/webtools/Venn/
