# Supplementary material for: Different responses of the rhizosphere microbiome to Verticillium dahliae infection in two cotton cultivars
Source: Front Microbiol. 2023 Aug 11;14:1229454. doi: 10.3389/fmicb.2023.1229454 (PMC10450913; doi:10.3389/fmicb.2023.1229454)
Supplement: Supplementary file 1 [file Data_Sheet_1.pdf]

Supplementary Material

Different responses of the rhizosphere microbiomes to *Verticillium dahliae* infection in two cotton cultivars

First Author*：Zhanjiang Tie[1], Peng Wang[2], Co-Author：XueKun Zhang[1]*, Hui Xi[1]*

*** Correspondence:**

Xuekun Zhang

Zhangxk2459@163.com

Hui Xi

xihui101101@126.comSupplementary Data

Supplementary Figures

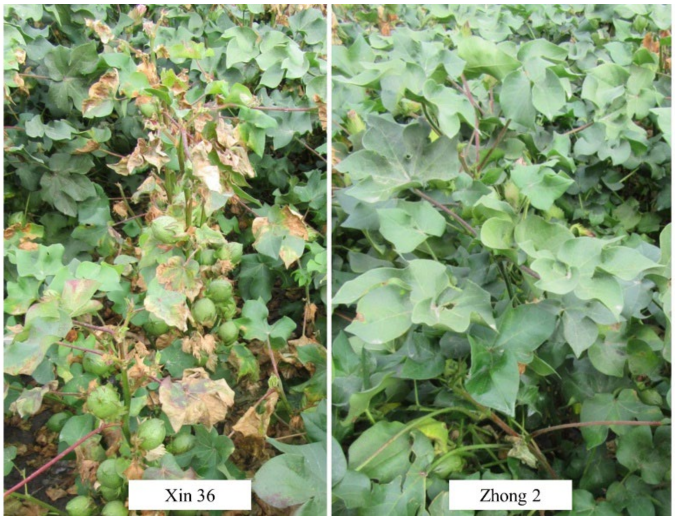

Supplementary Figure 1. Symptoms of Zhong 2 and Xin 36 plants infected by *V. dahliae* in the nursery of cotton Verticillium wilt identification.

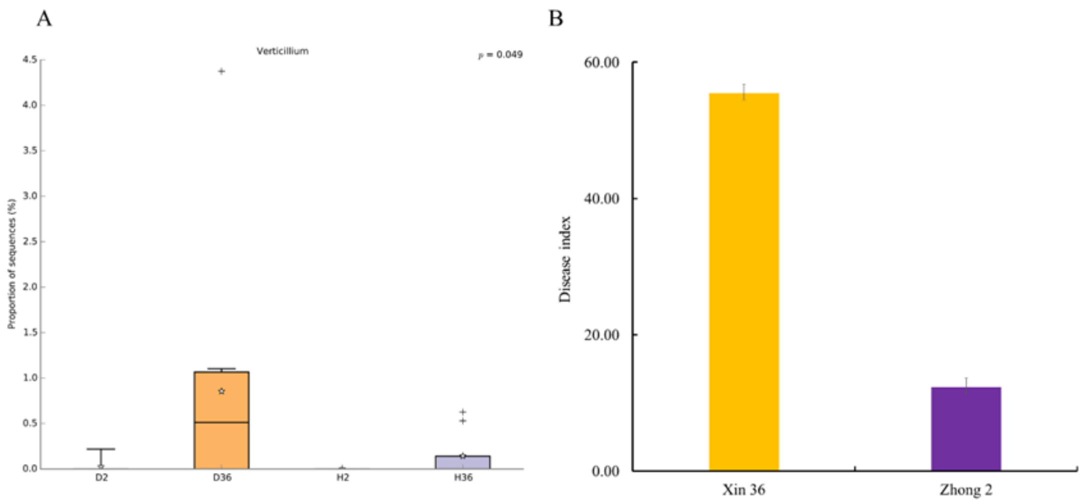

Supplementary Figure 2. Different responses of two cotton varieties to *V. dahliae* infection. A. The relative abundance of *Verticillium* in healthy and diseased rhizospheres of two cultivars. B. Investigation results of two cultivars in the nursery of cotton Verticillium wilt identification.

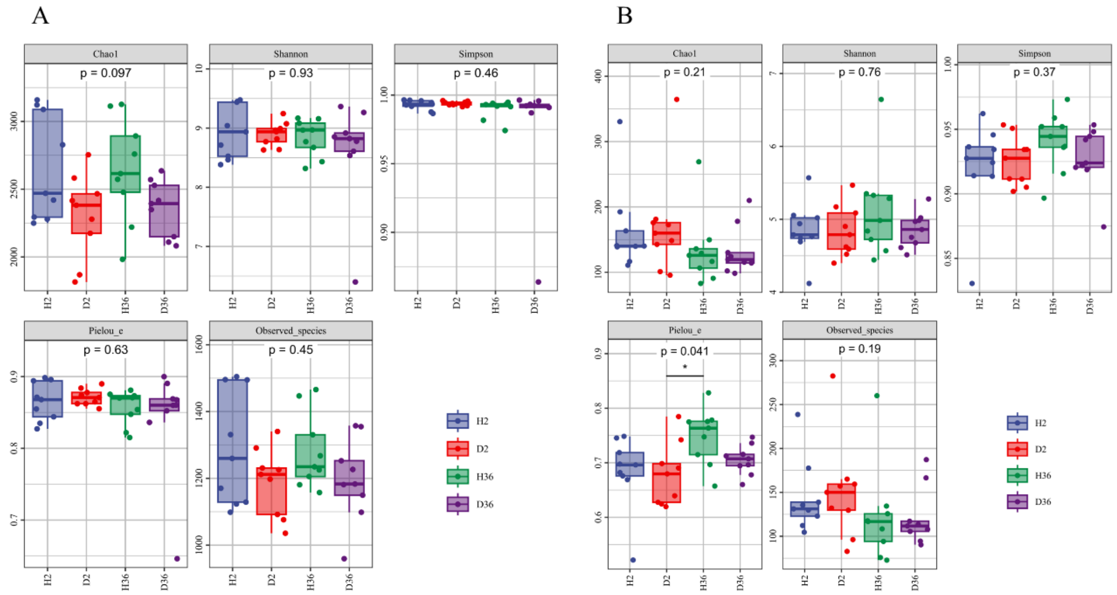

Supplementary Figure 3. Alpha diversity of healthy and diseased rhizosphere microbiome in two cotton cultivars.
